# Exercise habits and musculoskeletal health of the Finnish Dachshund population: an online survey study

**DOI:** 10.1186/s13028-026-00877-2

**Published:** 2026-08-13

**Authors:** Kami Turunen, Anu Katriina Lappalainen, Jouni Juho Tapio Junnila, Heli Katariina Hyytiäinen, Liisa Iiris Onerva Lilja-Maula

**Affiliations:** 1 Department of Equine and Small Animal Medicine, Faculty of Veterinary Medicine, University of Helsinki, Viikintie 49, P.O. Box 57, FI-00014 Helsinki, Finland; 2 EstiMates OY, Turku, Finland

**Keywords:** Angular limb deformity, Chondrodysplasia, Chondrodystrophy, Conformation, Pes varus

## Abstract

**Background:**

Concern about conformation-related welfare issues in pedigree dogs has risen in recent years. The Dachshund is an example of a short-limbed, i.e. chondrodysplastic, dog breed. Chondrodysplasia may result in angular limb deformities such as elbow incongruity or hind limb growth disorder pes varus. However, the consequences of short-limbed structure on exercise habits or mobility have not been extensively investigated in this breed before. The aim of our study was to determine owner-perceived exercise habits, musculoskeletal health, limb conformations, and prevalence of pes varus in the Finnish purebred Dachshund population by using an online survey for data collection.

**Results:**

The majority of the dogs (75.8%, *n* = 485/640) exercised for one to three hours each day, of with intense exercise accounting for less than two hours for most dogs (88.7%). Musculoskeletal disorders were reported by 17.9% of owners. Pes varus (6.9%) was the most frequent musculoskeletal disorder, followed by front limb growth disorders (5.8%). More than half of the dogs (54.6%) had an abnormal angular conformation in one or more limbs. Dogs with bilateral carpal and/or tarsal valgus malposition (*P* < 0.001) were more likely to have less exercise and be less willing to locomote than dogs without these conditions. Mobility was likely to be decreased in dogs with any musculoskeletal disorder (*P* < 0.001) or any limb malposition (*P* = 0.007).

**Conclusions:**

We demonstrated that musculoskeletal disorders and abnormal limb conformations related to chondrodysplastic structure are common in Dachshunds and are associated with decreased amounts of exercise and impaired mobility in this otherwise active breed. Because of these welfare problems, healthier breed standards and breeding towards more normal limb conformation are warranted.

**Supplementary Information:**

The online version contains supplementary material available at https://doi.org/10.1186/s13028-026-00877-2.

## Background

The Dachshund is a dog breed known for its short-limbed appearance, also referred to as chondrodysplasia or disproportional dwarfism [1–3]. Many chondrodysplastic breeds were originally developed for tracking and underground hunting as well as for herding, where low stance was clearly advantageous. Hence, the short-limbed phenotype, being a desirable trait, has become fixed in several dog breeds [4–7]. Chondrodysplastic breeds are nowadays hugely popular among pet dogs. In a UK study consisting of over 1.5 million dogs, more than half of the dogs were classified as short-limbed [8]. Moreover, Dachshunds are one of the most popular short-limbed dog breeds internationally [9–12]. In Finland, the wire-haired standard Dachshund was the eleventh most popular breed in 2024, and if all nine Dachshund breed varieties are combined, the Dachshund is the second most popular dog breed in the country, with approximately 2,000 yearly registrations [13].

Short limbs, however, come with their own set of unwanted conditions. In a study by Ascher et al. (2009), miniature Dachshunds were reported to have several conformation related disorders [14]. One example is a larger back length to wither height ratio, which has been shown to increase the risk of intervertebral disc extrusion in miniature Dachshunds [15]. The Fédération Cynologique Internationale (FCI) breed standard states that the general appearance of a Dachshund is low, short-legged and elongated, but cow hocks, bowlegs, or feet that turn markedly inwards or outwards are considered severe faults [5]. Nevertheless, chondrodysplasia results phenotypically in limbs that are both short and curved, which is, when exaggerated, characteristic of angular limb deformities (ALDs) [16]. An angular limb deformity, like elbow incongruity, is most often seen in the front limbs, but also hind limb deformities, such as pes varus, exist. Pes varus, which in dogs is defined as medially curved distal tibia leading to a bow-legged appearance [17, 18], is reported especially in Dachshunds and is thought to be hereditary [17]. Surgical correction of the most severe forms of pes varus in Dachshunds has been described in the literature, but very little is known about its prevalence or impact on affected dogs’ daily lives [17–22]. What is known is that both pes varus and elbow incongruity may cause serious welfare issues such as discomfort, lameness, and lateral patellar luxation [16–23]. In addition, many chondrodysplastic breeds, including Dachshunds, are also chondrodystrophic, meaning they have an increased risk for intervertebral disc disease due to early degeneration of intervertebral discs [1, 2].

Mobility, defined as an ability to move without restriction or pain, is one of the most important elements affecting dogs’ quality of life (QoL) [24]. Breed-specific anatomy, such as short-limbed conformation, and related diseases can influence mobility [24]. Various questionnaires, including the Canine Orthopedic Index, Helsinki Chronic Pain Index, and Liverpool Osteoarthritis in Dogs, have been developed to measure dogs’ mobility [25–28]. These questionnaires have questions concerning lifestyle, attitude, function, exercise, gait, stiffness, effects of weather, and QoL [29]. These tools can be used to quantify factors, such as perceived pain and QoL, and allow veterinarians to, for example, assess the consequences of various musculoskeletal disorders (MSDs) or the effectiveness of a treatment [29].

Welfare issues in pedigree dogs, especially associated with extreme conformation, have gained vast public awareness in recent years. In a German survey study analyzing people’s perceptions on breed-related welfare problems, participants identified the Dachshund as a breed with extreme conformation linked to serious health problems [30]. A comprehensive survey investigating potential risk factors for intervertebral disc disease in Dachshunds has already examined lifestyle, such as exercise habits, but also conformation [31]. However, studies assessing the impact of ALDs or other MSDs on mobility or QoL in chondrodysplastic breeds are sparse. Therefore, new studies are needed to identify how musculoskeletal diseases, especially ones related to short-limbed conformation, affect the well-being of chondrodysplastic dog breeds.

The aim of our study was, by using an online survey, to determine owner-perceived exercise habits, mobility, and musculoskeletal health in the Finnish purebred Dachshund population. We also examined the prevalence of pes varus deformity and determined the limb conformations that are most typical for Dachshunds.

## Methods

### Study population

The target population consisted of all owners of Finnish purebred Dachshunds aged one year or more. Owners were asked to fill in a separate survey for each of their dogs that met the participation criteria. For dogs with multiple owners, the owners were asked to fill in the questionnaire together and only once for each dog. All respondents were required to be at least 15 years old.

 Ethical approval for this study was granted by the Research Ethics Committee on Animal Research of the University of Helsinki (Statement 10/2022). The data were subject to the European Data Protection Regulation (GDPR). Participation was voluntary and responses were kept confidential. Owner approval was confirmed by submission of survey answers.

### Questionnaire design and distribution

An electronic online questionnaire (REDCap online survey and database application, Vanderbilt University, Nashville, TN, USA) was used for data collection and storage. Questionnaire development was based on a literature review of factors related to musculoskeletal health and physical activity in dogs, with a particular focus on Dachshunds. The draft questions were subsequently evaluated by external experts (a veterinary orthopedist, a survey expert, and two physiotherapists) to ensure they adequately reflected the musculoskeletal health and physical activity in Dachshunds. Following expert review, the questionnaire was pilot tested with the target population (six Dachshund owners) to assess clarity and answerability, and final modifications were made accordingly.

The survey was in Finnish. The translated questionnaire is provided in Additional file 1. Topics of the survey were the owner’s contact information, the dog’s signalment, purpose, and general health status, and questions related to the dog’s exercise habits, mobility, musculoskeletal system, and limb conformations. The survey consisted of multiple-choice single-answer questions (27), open-ended questions (24), dichotomous questions (15), multiple-answer questions (7), and single-choice questions with only one possible answer option (3). The number of questions available for each respondent varied from 43 to 76 questions, depending on the answers given. Some questions could be left unanswered, and thus, sample size varied across the questions. The results of open-ended answers were evaluated and grouped for analysis according to the similarity of their content. When duplicates were encountered, the record with a less complete response was excluded. Survey responses where the owner’s consent to participate, age, or contact information was missing or the dog’s age, sex, or breed variety was not reported correctly were labelled as non-informative responses and excluded from the analysis.

The participants were recruited using advertisements in social media such as the Veterinary Teaching Hospital Facebook pages and breed-related Facebook groups, the Finnish Kennel Club’s and Finnish Dachshund Club’s websites, breed club magazine, and email directed to all Finnish Dachshund breeders. When the survey was advertised, it was described to be part of a larger pes varus-related research project.

The survey was accessible on mobile and desktop devices from 9 August 2022 to 30 September 2023.

### Scoring of exercise habits and musculoskeletal health

Two kinds of scores were used in our statistical analysis to better represent exercise habits and musculoskeletal health of the dogs. The exercise score (ES) was created to estimate the amount of exercise each dog receives regularly and how willing it is to locomote. The ES was formed based on owners’ responses to three different multiple-choice single-answer questions specified in Table 1. After combining all answers, each dog received a final score (range: 3–14 points). Higher ES score correlated with higher physical activity.

**Table 1:** Formation of Exercise Score (ES) and number of dogs in each category

Survey questions selected to form ES	Answer options and points received					
	1 point	2 points	3 points	4 points	5 points	Points min-max
How much exercise^a^ does your dog get daily?	Less than 30 min per day	At least 30 min per day	At least 1 h per day	At least 2 h per day	More than 3 h per day	1–5
	(*n* = 9/640)	(*n* = 70/640)	(*n* = 248/640)	(*n* = 237/640)	(*n* = 76/640)	
How much intense exercise^b^ does your dog get daily?	Less than 30 min daily	At least 30 min per day	At least 1 h per day	At least 2 h per day	More than 3 h per day	1–5
	(*n* = 162/639)	(*n* = 215/639)	(*n* = 190/639)	(*n* = 59/639)	(*n* = 13/639)	
Does your dog show reluctance to locomote?	Daily	Weekly	Occasionally	Not at all		1–4
	(*n* = 14/640)	(*n* = 8/640)	(*n* = 133/640)	(*n* = 485/640)		
Final ES^c^						3–14

^a^Daily exercise was defined as total amount of exercise such as walking, outdoor activity, play, and hobbies

^b^Intense exercise was defined as all active exercise such as energetic play, running, active hobbies, and brisk walks

^c^Higher score indicates more physical activity

The musculoskeletal ability score (MAS) was aimed at assessing the number of signs of dysfunction related to the musculoskeletal system. The MAS summed up owners’ responses to three different multiple-choice select-one questions specified in Table 2. After combining all answers, each dog received an MAS score (range: 3–12 points). Higher MAS score indicated less signs of dysfunction related to the musculoskeletal system, i.e. increased mobility.

**Table 2:** Formation of Musculoskeletal Ability Score (MAS) and number of dogs in each category

Survey questions selected to form MAS	Answer options and points received				
	1 point	2 points	3 points	4 points	Points min-max
Does your dog have a reluctance to jump?	Daily (*n* = 11/639)	Weekly (*n* = 7/639)	Occasionally (*n* = 92/639)	Not at all (*n* = 529/639)	1–4
Does your dog have difficulty getting up from a lying position?	Daily (*n* = 0/640)	Weekly (*n* = 0/640)	Occasionally (*n* = 11/640)	Not at all (*n* = 629/640)	1–4
Does your dog limp?	Daily (*n* = 7/640)	Weekly (*n* = 5/640)	Occasionally (*n* = 66/640)	Not at all (*n* = 562/640)	1–4
Final MAS^a^					3–12

^a^ Higher score indicates increased mobility

### Limb conformation assessments

**Fig. 1:**
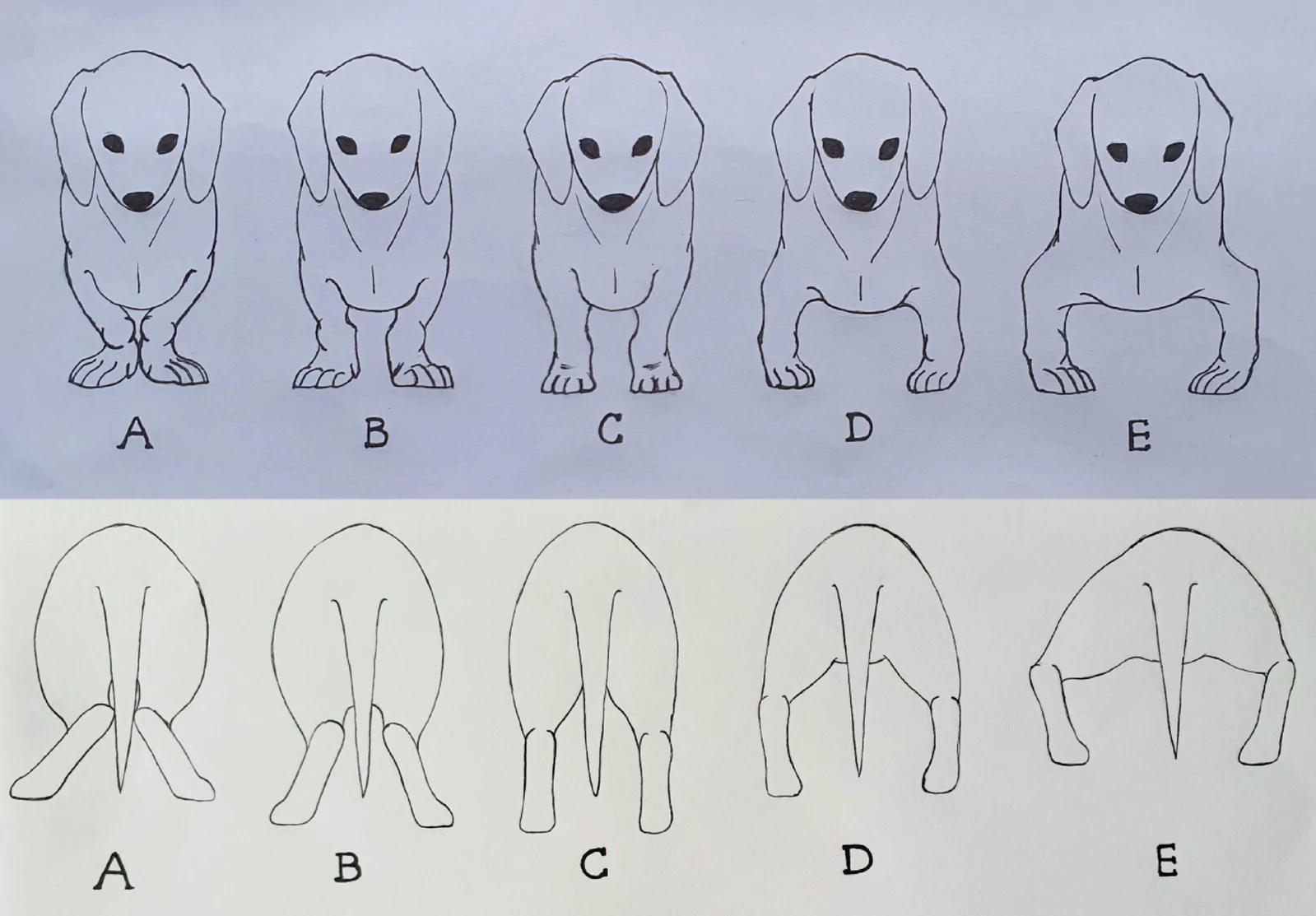
**S**urvey image scales for limb conformation assessments. **A** severe carpal (front limbs) or tarsal (hind limbs) valgus with external rotation of the distal limb. **B **moderate carpal or tarsal valgus with external rotation of the distal limb. **C** straight to mild changes. **D** moderate carpal or tarsal varus with internal rotation of the distal limb. **E** severe carpal or tarsal varus with internal rotation of the distal limb

Owners were asked to assess their dogs’ limb conformation based on image scales (Fig. 1) offered for each respondent. A series of five images of different limb conformations were provided for both front and hind limbs. Conformations varied from severe carpal or tarsal valgus with external rotation of distal limb to severe carpal or tarsal varus with internal rotation of distal limb. The image scale was developed to include limb conformations typical for front and hind limb ALDs found in Dachshunds, namely external rotation of the distal front limb and carpal valgus, and tarsal varus of the hind limbs [17, 32]. Owners chose the front and hind limb conformations best corresponding to their own dog’s limb structure. The assessment of the dog’s limb conformation was instructed to be made visually while the dog was standing and/or moving or by palpating the limbs. Each answer was marked as a letter, ranging from A-E, which correlated with the limb structure (Fig. 1). All carpal/tarsal valgus malpositions are later referred to simply as valgus, and all carpal/tarsal varus malpositions as varus.

### Statistical analysis

After the online survey was closed, data were downloaded from REDCap and exported into Excel^®^ spreadsheet, version 2505 (Microsoft, Redmond, WA, USA) for editing. Statistical analyses were conducted using SAS,^®^ version 9.4 (SAS Institute Inc., Cary, NC, USA). Two-sided P-values < 0.05 were considered statistically significant. Background variables chosen for the analysis were age, sex, neuter status, breed variety (standard vs. miniature), weight, primary intended purpose, number of dogs in household, acute and chronic diseases, medications/treatments, owner-reported QoL-impairing physical issues, any or specific MSDs (i.e., hip dysplasia, patellar luxation, front limb growth disorder, pes varus), heart diseases, intervertebral disc herniation, and limb conformations. Intervertebral disc herniation was classified as a neurological condition and analyzed separately from MSDs.

The potential effects of the selected background variables on the ES score were, depending on the type of background variable, first assessed with one-way Analysis of Variance or linear regression analyses. Secondly, variables shown to be statistically significant in the univariate analyses were included in a multivariate linear model. However, some of these variables (variables related to limb malposition, heart disease – any chronic disease, hip dysplasia – all MSDs) were strongly associated with each other. Distinguishing the effects of these strongly correlated pairs of variables is mathematically challenging – potentially causing unreliable results. Thus, a further simplified multivariate linear model was fitted, including only one variable of each of the mentioned variable-pairs. This was considered as the final statistical model. The effects were quantified using least square means and their 95% confidence intervals.

The MAS score was categorized into three categories (High: 12, Medium: 11, and Low: 10 or less) due to the low number of observations with MAS score < 10. The potential effects of the selected background variables on the MAS score were assessed with cumulative logit models. Similarly, as with the ES score, the variables were first evaluated with univariate models and in the second stage variables showing statistical significance (*P* < 0.01) in the univariate analyses were included in a multivariate cumulative logit model. A tighter cut-off value for the P-values was selected due to the large number of variables with P-value < 0.05. The models were constructed to estimate the probability to have higher MAS categories. The differences were quantified using odds ratios and their 95% confidence intervals.

The potential effects of the selected background variables on presence of chronic diseases, presence of owner-reported physical issues impairing QoL, and medications (yes/no) were assessed with logistic regression models. The potential effects of the selected background variables on time spent outdoors, number of walks, and running off-leash were assessed with cumulative logit models. For primary purpose, multinominal logit models were constructed to assess the potential effect of the background variables. Similarly, as presented for ES score, the variables were first evaluated with univariate models, and in the second stage variables showing statistical significance in the univariate analyses were included in multivariate models. The differences were quantified using odds ratios and their 95% confidence intervals.

## Results

### Study population

We received a total of 747 survey responses. After excluding all answers that did not meet our inclusion criteria, the final study population comprised 640 purebred Dachshunds, 58.4% (*n* = 374/640) females and 41.6% (*n* = 266/640) males. Of these, 79.2% (*n* = 507/640) were intact and 20.8% (*n* = 133/640) were neutered. The most frequent size variant was the standard Dachshund (58.1%, 372/640), followed by the miniature Dachshund (41.9%, 268/640). The median (range) age and weight were 3.8 (1–15.8) years and 8.0 (2.8–15.1) kg, respectively. Equal number of dogs came from multiple-dog households with four or more dogs (29.5%) and from single-dog households (29.4%), followed by two-dog households (26.9%), and three-dog households (14.2%). Additional features of the population demographics are presented in Additional file 2.

### Exercise habits

The majority of the dogs (87.7%, *n* = 561/640, Table 1) exercised for at least an hour each day, with the time varying from one to three hours for most dogs (75.8%, *n* = 485/640). For almost all dogs (88.7%, *n* = 567/639, Table 1), intense exercise accounted for less than two hours of the total amount of daily exercise, and more than half (59.0%, *n* = 377/639) exercised intensively for less than one hour each day.

All owners reported that their dog went out at least once a day, and most dogs (95.2%, *n* = 609/640) went out three times or more per day, with “going out” defined as all time spent outdoors, including both passive outdoor time and active exercise. The time spent outdoors was comparable to the overall exercise amounts as nearly all dogs (91.5%, *n* = 584/638) spent an hour or more outdoors each day. Older dogs were more likely to spend less time outdoors than younger dogs (*P* < 0.001). In addition, dogs that had heart disease (*P* = 0.024) or any kind of valgus malposition (*P* = 0.005) tended to spend less time outdoors than dogs without these conditions. In contrast, in households with four dogs or more, the dogs spent more time outdoors than dogs from households with fewer dogs. This was most evident when comparing households of one or two dogs to households of four or more dogs (*P* < 0.001, Additional file 3). All factors affecting the time spent outdoors can be found in Table 3.

**Table 3:** Factors affecting exercise habits

	Odds ratio	95% CI		T-value	*P*-value
		Lower	Upper		
Less time spent outdoors					
Valgus malposition^a^: Yes vs. No	1.54	1.14	2.08	2.81	0.005^*^
Heart disease: Yes vs. No	3.36	1.17	9.63	2.26	0.024^*^
Number of dogs^b^: Single vs. Multiple	1.56	1.11	2.21	2.55	0.011^*^
Age: One year increase in age	1.11	1.05	1.17	3.92	< 0.001^*^
Fewer walks^c^					
Patellar luxation: Yes vs. No	2.23	1.02	4.90	2.01	0.045^*^
Number of dogs: Single vs. Multiple	0.44	0.31	0.64	-4.41	< 0.001^*^
Age: One year increase in age	1.09	1.04	1.14	3.77	< 0.001^*^
Less running off-leash					
Number of dogs: Single vs. Multiple	4.22	2.89	6.16	7.45	< 0.001^*^

^a^Carpal and/or tarsal valgus malposition in any limb(s)

^b^Number of dogs living in the same household

^c^At least 15 min of brisk walking on a leash or off-leash

^*^Statistically significant result

Daily walks, defined as at least 15 min of brisk walking on a leash or off-leash, were done with almost all dogs (93.4%, *n* = 598/640, Additional file 4). Several factors were associated with the number of walks (Table 3), such as the number of dogs in the household and the existence of patellar luxation. Dogs living in a household with fewer dogs were likely to receive more walks, and this was especially evident in households of one dog compared with households of four or more dogs (*P* < 0.001, Additional file 3). Dogs with patellar luxation (*P* = 0.045) and dogs of older age (*P* < 0.001) were likely to have less walks than dogs without patellar luxation or younger dogs.

Almost every owner let their dog run off-leash–defined here as running without a leash in, for example, a yard, enclosure, or forest–at least occasionally (98.0%, *n* = 627/640, Additional file 4), and more than half reported doing this daily (57.0%, *n* = 365/640). Dogs from multi-dog households were more likely to be running off-leash than dogs from single-dog households (Table 3). The difference was most apparent between households of one dog and households of four or more dogs (*P* < 0.001, Additional file 3).

Most typical surfaces used for outdoor activities were dirt road (70.1%, *n* = 447/638), forest terrain or similar (69.6%, *n* = 444/638), and grass, field, or similar (62.4%, *n* = 412/638), followed by asphalt or similar (57.5%, *n* = 367/638).

Almost all dogs (98.7%, *n* = 629/637) engaged in at least one physical activity-based hobby with their owners, the most frequent hobbies being walking on a leash or off-leash (94.8%, *n* = 604/637), hunting activities (46.3%, *n* = 295/637), obedience and/or rally obedience (7.2%, *n* = 46/637), running and/or canicross (6.3%, *n* = 40/637), and nosework (4.1%, *n* = 26/637). Physical play was also common among Dachshunds, although a small portion of owners reported that their dog did not play at all (4.5%, *n* = 29/638). The most common forms of physical play included running off-leash (75.9%, *n* = 484/638), playing with toys (70.2%, *n* = 448/638), playing with other dogs (66.0%, *n* = 421/638), digging (44.2%, *n* = 282/638), and fetching (42.8%, *n* = 273/638).

**Fig. 2:**
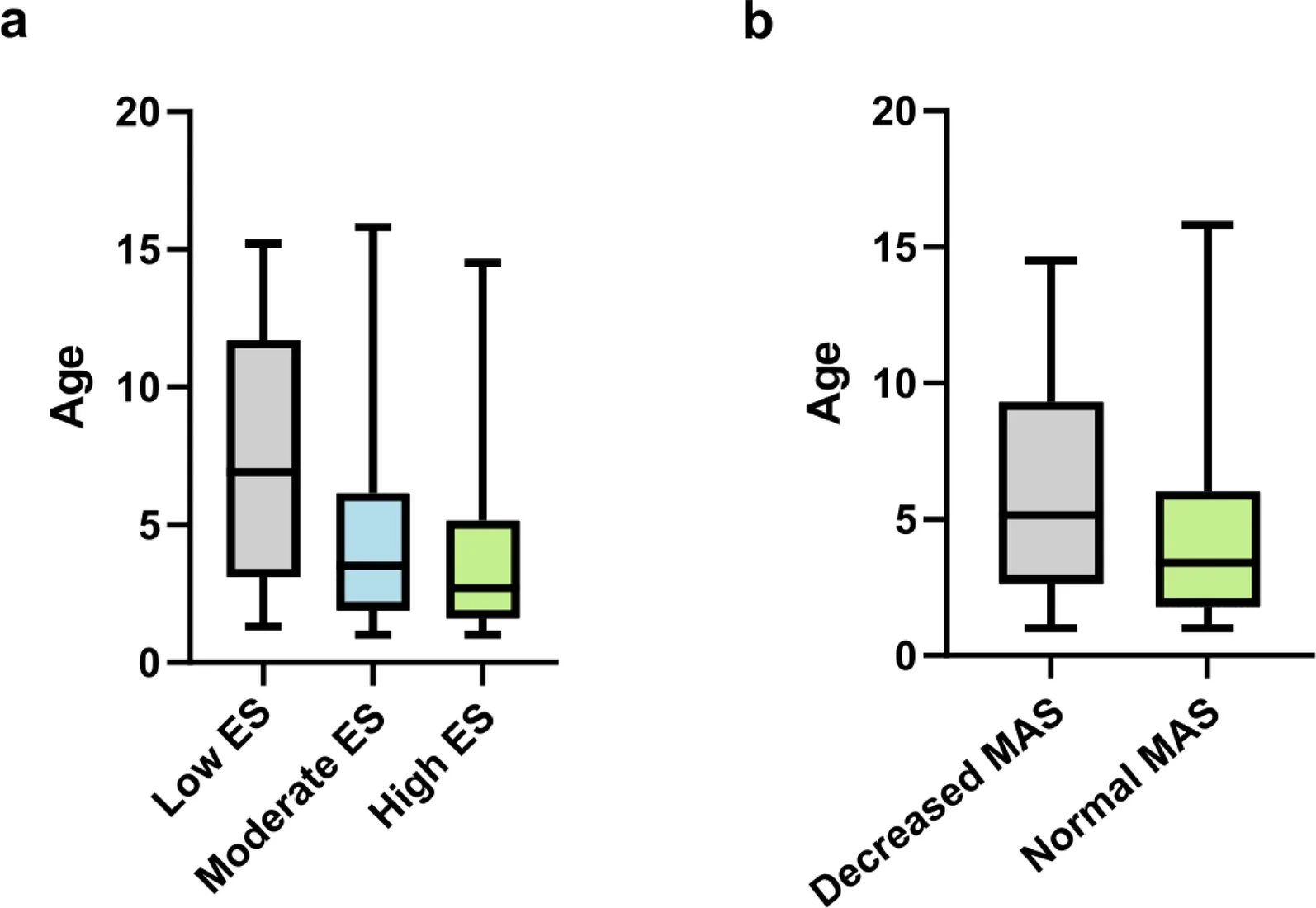
Box plot showing exercise score and musculoskeletal ability score relative to age. The box represents the interquartile range (Q1–Q3), the line in the box indicates the median, and whiskers represent the minimum and maximum values. **a** ES=exercise score (3–14 points), low ES defined as 3–7 points, moderate ES 8–10 points and high ES 11–14 points; higher score indicates more physical activity. **b** MAS=musculoskeletal ability score (3–12 points), decreased MAS defined as 3–11 points indicating dog shows any signs related to dysfunction of musculoskeletal system, and normal MAS score defined as 12 i.e. the dog does not show signs of musculoskeletal dysfunction

Several background variables, such as sex and limb conformation, were found to be associated with the exercise score (Table 4). Dogs with bilateral valgus malposition in front and/or hind limbs (*P* < 0.001) or hip dysplasia (*P* = 0.038) were more likely to have lower ES than dogs without these conditions. Female dogs (*P* < 0.001) and older dogs (*P* < 0.001, Fig. 2a) had lower ES than males and younger dogs. Also, the number of dogs in the household affected the ES. Dogs living in a household without other dogs had the lowest ES, households with four dogs or more had the highest ES, and this difference was particularly evident when comparing these two groups to each other (*P* < 0.001, Additional file 3).

**Table 4:** Factors affecting Exercise Score (ES)

	ES estimate^a^	95% CI		*P*-value
		Lower	Upper	
Bilateral valgus^b^: Yes vs. No	-0.5077	-0.8009	-0.2146	< 0.001^*^
Hip dysplasia: Yes vs. No	-0.9543	-1.8560	-0.0526	0.038^*^
Number of dogs^c^: Single vs. Multiple	-0.6765	-1.0098	-0.3433	< 0.001^*^
Sex: Female vs. Male	-0.5581	-0.8511	-0.2652	< 0.001^*^
Age: One year increase in age	-0.1699	-0.2173	-0.1226	< 0.001^*^

^a^Higher ES indicating more physical activity

^b^Bilateral carpal and/or tarsal valgus malposition

^c^Number of dogs living in the same household

^*^ Statistically significant result

### Overall and musculoskeletal health

Out of all dogs, 6.3% (*n* = 40/640) had some sort of physical issue that impaired their daily QoL, with musculoskeletal (61.5%, *n* = 24/39) and cardiovascular (15.4%, *n* = 6/39) conditions being the most common. Any varus malposition in the hind or front limbs (*P* = 0.004), being neutered (*P* = 0.041), and increasing age (*P* < 0.001) were found to be risk factors for owners reporting QoL impairing issues (Table 5).

**Table 5:**
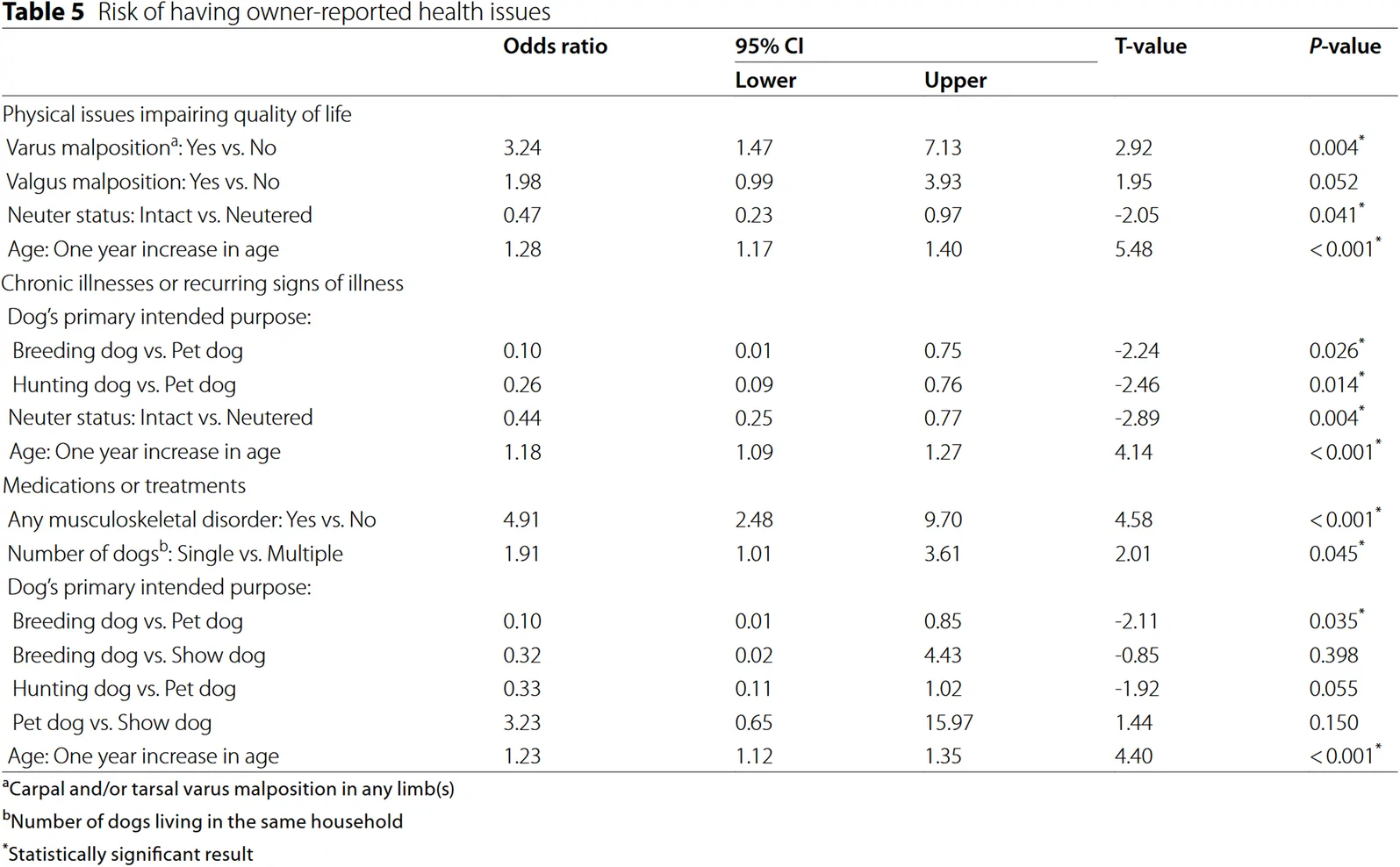
Risk of having owner-reported health issues

^a^Carpal and/or tarsal varus malposition in any limb(s)

^b^Number of dogs living in the same household

^*^Statistically significant result

Chronic illnesses or recurring signs of illness were reported in 11.7% (*n* = 75/640) of dogs, the most common causes being immunological (32.4%, *n* = 24/74), musculoskeletal (24.0%, *n* = 18/74), and cardiovascular (16.2%, *n* = 12/74). The dog’s primary intended purpose being a pet increased the odds of chronic illnesses compared with breeding dog (*P* = 0.026) or hunting dog (*P* = 0.014). In addition, both neuter status and age were associated with chronic illnesses. Neutered dogs (*P* = 0.004) and older dogs (*P* < 0.001) were more likely than intact and younger dogs, respectively, to have chronic illnesses (Table 5).

Health issues (65.2%, *n* = 45/69), such as problems related to poor conformation, and temperament (33.3%, *n* = 23/69), e.g. lack of prey drive, were reported to be the two main reasons for owners to state that their dog was not suitable for its intended purpose (10.8%, *n* = 69/640). The primary intended purpose of dogs was associated with multiple factors (Additional file 5). For example, dogs with any limb malposition were more likely to have been acquired as pets than for breeding (*P* = 0.016) or show (*P* = 0.006) purposes. Similarly, in single-dog households, dogs were most likely acquired as pets rather than for other purposes, particularly when compared with households of four or more dogs (*P* < 0.001).

A tenth of dogs (9.8%, *n* = 63/640) were reported to be given medications and/or to receive some sort of treatment. Several factors, such as higher age (*P* < 0.001) and any MSD (*P* < 0.001), were found to increase the likelihood of this (Table 5).

Changes in mobility and willingness to locomote were reported regularly, with reluctance to locomote being the most common type of change (24.2%, *n* = 155/640), followed by reluctance to jump (17.2%, *n* = 110/639), lameness (12.2%, *n* = 78/640), and difficulty getting up from a lying position (1.7%, *n* = 11/640). Weather (41.6%, *n* = 64/154), such as hot, cold or rainy weather, was the single most frequently reported reason for reluctance to locomote, health being the second (37.7%, *n* = 58/154), and behavioral factors, such as temperament and motivation, the third (35.1%, *n* = 54/154). In addition, 69.5% (*n* = 105/151) of dogs that were reported to have reluctance to locomote also had an abnormal angular conformation in one or more limbs.

At least one MSD was reported in 17.9% (*n* = 114/637) of dogs. Pes varus (6.9%, *n* = 44/639) was the most typically reported MSD, followed by front limb growth disorders (5.8%, *n* = 37/640), patellar luxation (3.1%, *n* = 20/640), and hip dysplasia (2.3%, *n* = 15/640). Only a small number of dogs (2.7%, *n* = 17/640) had received treatment for hind or front limb growth disorders, surgery being the most frequent treatment option (64.7%, *n* = 11/17).

The prevalence of intervertebral disc herniation was 5.2% (*n* = 33/640) and the median age (range) at first signs was 4.8 years (2–12 years). The most common signs related to intervertebral disc herniation were pain and/or reluctance to locomote (81.8%, *n* = 27/33), front and/or hind limb paresis or ataxia (33.3%, *n* = 11/33), and front and/or hind limb paralysis (24.2%, *n* = 8/33).

Several factors affecting the musculoskeletal ability score were found (Table 6). The MAS was likely to be lower in dogs with any MSD (*P* < 0.001), any limb malposition (*P* = 0.007), or intervertebral disc herniation (*P* = 0.009) than in dogs without these disorders. Also, male dogs (*P* < 0.001) and older dogs (*P* = 0.002, Fig. 2b) were more likely to have lower MAS than female dogs or younger dogs.

**Table 6:** Factors affecting Musculoskeletal Ability Score (MAS)

	Odds ratio^a^	95% CI		T-value	*P*-value
		Lower	Upper		
Any musculoskeletal disorder: Yes vs. No	0.41	0.26	0.67	-3.60	< 0.001^*^
Any malposition^b^: Yes vs. No	0.55	0.35	0.85	-2.70	0.007^*^
Intervertebral disc herniation: Yes vs. No	0.38	0.18	0.79	-2.60	0.009^*^
Physical issues impairing quality of life: Yes vs. No	0.26	0.12	0.55	-3.50	< 0.001^*^
Sex: Male vs. Female	0.48	0.32	0.73	-3.45	< 0.001^*^
Age: One year increase in age	0.90	0.85	0.96	-3.05	0.002^*^

^a^Probability to be in a higher ordered MAS group, i.e. to have better mobility

^b^Carpal and/or tarsal varus and/or valgus malposition in one or more limbs

^*^Statistically significant result

### Limb conformation

More than half of the owners reported their dog (54.6%, *n* = 344/630) to have some sort of abnormal angular conformation in one or more limbs, more commonly in the front limbs (41.7%, *n* = 263/631) than in the hind limbs (31.7%, *n* = 200/630). These types of malposition are illustrated in Fig. 1 (A–E), where panels A and E represent severe valgus/varus and panels B and D moderate valgus/varus. Valgus malposition was reported in 38.3% (*n* = 483/1,262) of front limbs and in 16.5% (*n* = 208/1,260) of hind limbs. Valgus was moderate in 96.3% (*n* = 465/483) and severe in 3.7% (*n* = 18/483) of front limbs, and moderate in 99.0% (*n* = 206/208) and severe in 1% (*n* = 2/208) of hind limbs with reported valgus malposition. Carpal valgus was common within the group of dogs diagnosed with any front limb growth disorder (59.5%, *n* = 22/37). Varus malposition was nearly non-existent in front limbs (0.3%, *n* = 4/1,262) and uncommon in hind limbs (13.4%, *n* = 169/1,260). All varus cases were reported to be moderate in front limbs. Varus was moderate in 84.0% (*n* = 142/169) and severe in 16.0% (*n* = 27/169) of hind limbs with reported varus malposition. The highest proportion of tarsal varus was reported in the group of dogs suspected or diagnosed with pes varus (86.0%, *n* = 37/43).

## Discussion

This study explored dogs’ exercise habits, musculoskeletal health, and limb conformations as reported by Finnish Dachshund owners. It appeared that despite its lower stance and long back, the Dachshund is an active breed, widely used for various physically demanding activities such as hunting. However, MSDs and abnormal limb conformations were frequently reported and negatively affected daily exercise habits and mobility.

The amount of daily exercise varied considerably, but nearly 90% of Dachshunds exercised for at least an hour each day. This exceeds the amount reported in past surveys, where slightly less than 60% of dachshunds were active for more than an hour daily [31, 33]. However, the exercise amount in these studies was measured differently from ours, the main distinction being that they focused only on exercise occurring outside the house, and we included all forms of exercise in our study.Another reason for the high amount of physical activity reported in our study might have been that most Dachshunds came from multiple-dog households, although in several countries, including Finland, the most typical number of dogs per household is one [34–37]. The relatively high proportion of multi-dog households in our sample may be partly explained by the recruitment strategy, which included direct marketing to dog breeders. Breeders, who often own multiple dogs, are also likely to follow breed-specific communication channels more closely and may therefore have been more easily reached and more motivated to respond to the survey compared to single-dog households. We demonstrated that an increasing number of dogs living in same household increased the likelihood of dogs having more exercise, spending more time outdoors, being allowed to run off-leash more often, and being more willing to locomote than dogs in households with fewer dogs. This result is comparable to a previous study, where the activity levels of dogs left home alone on a regular basis were found to be higher in multi-dog households than in single-dog households, and this was speculated to be because of increased stimulation [38]. In addition, dogs from multi-dog households may be more likely to live in less densely populated areas and have access to their own yard and thus, have more opportunities to spend time outdoors and to exercise compared with dogs from single-dog households. Furthermore, the age of the dog is a significant factor influencing overall activity. Our study revealed that younger dogs are more likely to have more exercise, spend more time outdoors, go for more walks, and be more willing to locomote. These findings are consistent with research showing that increasing age decreases physical activity levels [39–41]. The Dachshunds in our study were mainly young adults, which likely contributed to the high exercise amounts reported by owners.

Interestingly, weather, such as cold or rainy weather, was the single most frequently reported reason for reluctance to locomote in our study. Effects of weather have previously been investigated in dogs. Results from a recent survey implied that activity levels of dogs weighing under 10 kg were more negatively affected by adverse weather than activity levels of larger dogs [33]. This could be partially explained by owners’ assumed tendency to provide more exercise to larger dogs regardless of weather conditions. Nevertheless, small size of the dog, because of a larger surface area-to-volume ratio, means greater heat loss. Thus, it has been suggested that small dogs need to devote a larger proportion of their metabolic energy to thermogenesis to offset increased heat loss [42]. This applies well to our study population, which consisted mainly of Dachshunds weighing 10 kg or less. Moreover, short limbs cause wide surface area with relatively thin insulation of hair to be situated in close proximity to the ground, where skin becomes exposed to extreme heat, wetness, or coldness of the given surface.

According to our data, medications and treatments were more common in dogs intended primarily as pets than in breeding dogs, and in single-dog households than in households with four or more dogs. Breeding typically requires dogs to be in good health; therefore, dogs with health issues may be less likely to be selected for breeding. In our study population, Dachshunds with any limb malposition were more likely to have been acquired as a pet than for breeding purposes. In addition, dogs from single-dog households were more often acquired as pets rather than for other purposes, whereas dogs from households with four or more dogs were more likely to be obtained for breeding than as pets. These findings might partly explain the higher prevalence of medications and treatments in pet dogs and in single-dog households.

Our data also suggests that owners were more likely to report chronic illnesses or physical issues impairing QoL in neutered Dachshunds. As the reason for neutering was not recorded in our sample population, it is possible that poor health led to neutering in some cases, which may partially explain these results. Nevertheless, neutering has been associated with an increased risk of certain joint disorders, cancers, and urinary incontinence in dogs [43–47]. However, breed-specific differences exist, and no increased risk of these conditions has been demonstrated in Dachshunds [46, 47]. On the other hand, studies investigating the effect of neutering on intervertebral disc disease have yielded inconsistent findings, with some reporting an association and others not [31, 48, 49]. Taken together, the overall impact of neutering on health in Dachshunds remains unclear and warrants further investigation.

Regarding musculoskeletal disorders, these were frequent in our study population, and they were reported as the main reason for impaired QoL. The nearly 18% prevalence observed in our study is higher than in previous studies, where the prevalence of MSDs in dogs of various breeds attending primary veterinary care was reported to be around 10% [50, 51]. One reason might be the short-limbed conformation of Dachshunds. The predominantly reported MSDs in our study were pes varus and front limb growth disorders, both of which are linked to the chondrodysplastic nature of this breed. Although the occurrence of pes varus has not been studied before, it is possible that the nearly 7% prevalence in our data is higher than the actual rate. Our survey was advertised as part of a larger pes varus-related research project, which may have led to selection bias and thus increased the proportion of such cases in our data. In addition, the question concerning this disease was phrased to include suspected cases as well, some of which may not represent true diagnoses.

An online health assessment tool that allows people to evaluate their dogs’ conformation, including limb alignment and height, was recently released for public use in the UK [52]. Using these kinds of tools, abnormal angular limb conformation could be detected by owners before other signs of illness, such as pain or lameness, appear. Body conformation measurements focusing on back-to-height ratios have already been collected from Dachshund owners in a survey [31], but owner-perceived limb conformations that emphasize limb alignment have not been previously investigated in this breed. According to our data, more than half of the Dachshunds had an abnormal angular limb conformation, with carpal valgus and external rotation of the distal front limb being the most common type of malposition. This is a large proportion of dogs, yet the true amount might be even higher since only the frontal plane conformation was assessed in our study, and milder changes might be difficult to recognize. An earlier study focusing on elbow incongruity implied that a milder form of a short radius or short ulna causes less pronounced external changes [53]. Notably, our findings suggest that moderate and severe angular limb conformations can have a negative impact on quantity of exercise, willingness to locomote, and mobility in Dachshunds. This is consistent with an earlier study showing that dogs with more carpal valgus and distal limb external rotation were more likely to exhibit lameness and joint pain [16]. Furthermore, it has been previously demonstrated that more severe frontal plane ALDs in the canine antebrachium have higher odds of showing any radiographically detectable joint disease [54].

Our findings warrant further studies focusing on various MSDs resulting from chondrodysplastic structure and on breeding strategies to reduce their harmful impact. For decades, there has been a radiographic screening method aimed at finding disc calcifications, yet no validated health screening tests are available for hind limb deformities such as pes varus [55]. More recently, a new radiographic screening technique was developed to assess elbow incongruity in short-limbed dog breeds, and it is currently used in Finland [23, 56]. New tests, such as goniometric measurements of front limb rotation and carpal valgus, have been created and may be used for assessing the severity of front limb conformational changes in the coming years [32]. Nevertheless, all conformational issues might not be solved using merely chondrodysplastic dogs for breeding since short limbs are the cause of these problems. Thus, the aim of breeding may need to shift towards not only straighter but also longer limb structure, and this requires alterations to the breed standards. Genetic analysis has revealed two retrogenes associated with short limb structure, namely FGF4 retrogene on chromosome 18 (FGF4L1) and 12 (FGF4L2) [3, 57]. Further studies have shown that copy numbers of these retrogenes reduce the limb length further, with FGF4L1 having much greater impact on limb length than FGF4L2, which is also associated with chondrodystrophy [57–59]. The Finnish Dachshund Club currently recommends screening dogs for FGF4L2 retrogene to enable the use of dogs carrying at least one normal allele in breeding and to increase their proportion in the population over time [60]. However, Dachshunds have high frequencies of both retrogenes, so there is little room for selection through breeding [61–63]. Fortunately, there is also the possibility of using breeding strategies focusing on introducing new genetic material such as cross-breeding or an open breed registry [64]. It is also important to acknowledge that owners do not always recognize MSDs as chronic illnesses, as there was a discrepancy between reported prevalence of chronic illnesses and MSDs in our study. Therefore, raising public awareness regarding the harmful consequences of short-limbed conformation is crucial.

The main limitations in our study derive from the subjective nature of surveys in general. The phrasing of questions in the questionnaire may lead to constraints on interpretations and thus the answers given by respondents. In addition, the failure of owners to recognize their dogs’ abnormal behavior, especially when related to health issues, can undermine the reliability of the results. Moreover, some of the limb conformations reported by owners might not present accurately the true conformation of the dogs because our image scale was constructed to include several conformational variations in one image. However, individual characteristics of ALDs may occur independently of each other [16]. Furthermore, the prevalence of intervertebral disc herniation and MSDs reported in our study can be underestimated, as the inclusion criteria required dogs to be alive, and severe cases may have resulted in euthanasia. Additionally, our sample consisted mainly of young dogs, some of which may still develop intervertebral disc herniation later in life [61, 65, 66]. Despite these shortcomings, our research highlights some of the threats posed by short-limbed structure to the health and well-being of Dachshunds.

## Conclusions

Our study demonstrates that owner-reported musculoskeletal disorders and abnormal limb conformations related to chondrodysplastic structure are common in Dachshunds. Abnormal angular limb conformations are associated with a decreased amount of exercise and impaired mobility in this otherwise active breed. Because of these welfare problems, healthier breed standards and breeding towards more normal limb conformation are warranted. Additionally, public awareness regarding the harmful consequences of short-limbed structure needs to be raised.

## Supplementary Information

Below is the link to the electronic supplementary material.

Supplementary Material 1. Translated questionnaire. Translation of the original Finnish online survey Mäyräkoirien liikuntakysely 2022 used for data collection.

Supplementary Material 2. Population demographics. ^a^ P_10_-P_90_ denote the 10th, 25th, 50th, 75th and 90th percentiles, respectively. ^b^ Both miniature and kaninchen size variations are included in miniature size

Supplementary Material 3. Associations between number of dogs in the household and exercise habits alongside medication usage. ^a^ Higher ES indicating more physical activity. ^b^ Number of dogs living in the same household. ^c^ At least 15 min of brisk walking on a leash or off-leash. ^*^ Statistically significant result

Supplementary Material 4. Exercise habits of the Finnish Dachshund population. Daily amount of total (a) and intense (b) exercise. c: number of daily walks, i.e. at least 15 min of brisk walking on or off-leash. d: graph indicates how often dogs are allowed to run off-leash

Supplementary Material 5. Factors associated with dog’s primary intended purpose. Odds ratios for differences between groups when other purposes are compared to Pet dogs. ^a^ Carpal and/or tarsal varus and/or valgus malposition in one or more limbs. ^b^ Number of dogs living in the same household. ^*^ Statistically significant result

## Data Availability

The datasets used and/or analyzed in this study are available from the corresponding author on reasonable request.

## Abbreviations

ALD: Angular limb deformity
ES: Exercise score
MAS: Musculoskeletal ability score
MSD: Musculoskeletal disorder
QoL: Quality of life

## Declarations

### Prior publication

The data have not been published previously.

## References

[1] Hansen HJ. A pathologic-anatomical study on disc degeneration in dog, with special reference to the so-called enchondrosis intervertebralis. Acta Orthop Scand Suppl. 1952;23:1–130. 10.3109/ort.1952.23.suppl-11.01 14923291

[2] Braund KG, Ghosh P, Taylor TKF, Larsen LH. Morphological studies of the canine intervertebral disc. The assignment of the beagle to the achondroplastic classification. Res Vet Sci. 1975;19:167–72. 1166121

[3] Parker HG, VonHoldt BM, Quignon P, Margulies EH, Shao S, Mosher DS, et al. An expressed FGF4 retrogene is associated with breed-defining chondrodysplasia in domestic dogs. Science. 2009. 10.1126/science.1173275. PMC2748762 19608863

[4] Bellamy KKL, Lingaas F. Short and sweet: Foreleg abnormalities in Havanese and the role of the FGF4 retrogene. Canine Med Genet. 2020. 10.1186/s40575-020-00097-5. PMC7720493 33372642

[5] Fédération Cynologique Internationale. FCI-Standard N° 148 Dachshund. 2022. https://www.fci.be/Nomenclature/Standards/148g04-en.pdf. Accessed 20 Mar 2025.

[6] Fédération Cynologique Internationale. FCI-Standard N° 38 Welsh Corgi (Cardigan). 2022. https://www.fci.be/Nomenclature/Standards/038g01-en.pdf. Accessed 30 Oct 2025.

[7] Fédération Cynologique Internationale. FCI-Standard N° 302 Irish Glen of Imaal Terrier. 2024. https://www.fci.be/Nomenclature/Standards/302g03-en.pdf. Accessed 30 Oct 2025.

[8] O’Neill DG, McMillan KM, Church DB, Brodbelt DC. Dog breeds and conformations in the UK in 2019: VetCompass canine demography and some consequent welfare implications. PLoS ONE. 2023. 10.1371/journal.pone.0288081. PMC10370710 37494312

[9] Inoue M, Hasegawa A, Hosoi Y, Sugiura K. Breed, gender and age pattern of diagnosis for veterinary care in insured dogs in Japan during fiscal year 2010. Prev Vet Med. 2015. 10.1016/j.prevetmed.2015.02.010. 25746927

[10] Sallander M, Hedhammar Å, Rundgren M, Lindberg JE. Demographic data of a population of Swedish dogs measured in a questionnaire study. Acta Vet Scand. 2001;42:71–80. 10.1186/1751-0147-42-71 PMC2202336 11455903

[11] Haid M. The most popular dog breeds of 2024. 2024. https://www.akc.org/expert-advice/news/most-popular-dog-breeds-2024. Accessed 20 Mar 2025.

[12] Kennel Club. Comparative tables of registrations for the years 2015–2024 inclusive. https://kc-media-production.azureedge.net/media/rdodkdnn/10-yearly-breeds-stats-hound.pdf. Accessed 20 Mar 2025.

[13] Finnish Kennel Club. KoiraNet Breeding Database. https://jalostus.kennelliitto.fi/frmRekisteroinnit.aspx?Lang=en. Accessed 14 Apr 2025.

[14] Asher L, Diesel G, Summers JF, McGreevy PD, Collins LM. Inherited defects in pedigree dogs. Part 1: disorders related to breed standards. Vet J. 2009. 10.1016/j.tvjl.2009.08.033. 19836981

[15] Packer RMA, Hendricks A, Volk HA, Shihab NK, Burn CC. How long and low can you go? Effect of conformation on the risk of thoracolumbar intervertebral disc extrusion in domestic dogs. PLoS ONE. 2013. 10.1371/journal.pone.0069650. PMC3722130 23894518

[16] Lappalainen A, Pulkkinen HSM, Mölsä S, Junnila J, Hyytiäinen H, Vapaavuori O. Breed-typical front limb angular deformity is associated with clinical findings in three chondrodysplastic dog breeds. Front Vet Sci. 2023. 10.3389/fvets.2022.1099903. PMC9887302 36733429

[17] Johnson SG, Hulse DA, Vangundy TE, Green RW. Corrective osteotomy for pes varus in the Dachshund. Vet Surg. 1989;18:373–9. 10.1111/j.1532-950x.1989.tb01103.x 2815554

[18] Radasch RM, Lewis DF, McDonald DE, Calfee EF, Barstad RD. Pes varus correction in Dachshunds using a hybrid external fixator. Vet Surg. 2008. 10.1111/j.1532-950X.2007.00350.x. 18199059

[19] Izumisawa Y, Seno T, Abe R, Miyoshi K, Maehara S, Wakaiki S, et al. Axial correction of pes varus by transverse-opening wedge osteotomy and T-plate fixation with beta-tricalcium phosphate (β-TCP) transplantation in Dachshunds. J Vet Med Sci. 2005;67:437–40. 10.1292/jvms.67.437 15876796

[20] Sellier C, Dosal MM, Guthrie JW, Fitzpatrick N. Correction of pes varus deformity in a miniature Dachshund by true spherical osteotomy with a dome saw blade. J Am Vet Med Assoc. 2020;257:624–30. 10.2460/javma.257.6.624 32857004

[21] Chau L, Wilson L. Pes varus correction in dachshunds with mini hybrid external skeletal fixators. Aust Vet J. 2022;100:135–45. 10.1111/avj.13139 34907526

[22] Petazzoni M, Nicetto T, Vezzoni A, Piras A, Palmer R. Treatment of pes varus using locking plate fixation in seven Dachshund dogs. Vet Comp Orthop Traumatol. 2012. 10.3415/VCOT-11-03-0035. 22286944

[23] Lappalainen AK, Hyvärinen T, Junnila J, Laitinen-Vapaavuori O. Radiographic evaluation of elbow incongruity in Skye terriers. J Small Anim Pract. 2016. 10.1111/jsap.12438. 26698951

[24] Ramos MT, Otto CM. Canine mobility maintenance and promotion of a healthy lifestyle. Vet Clin Small Anim. 2022. 10.1016/j.cvsm.2022.03.001. 35562216

[25] Brown DC. The Canine Orthopedic Index. Step 1: Devising the Items. Vet Surg. 2013. 10.1111/j.1532-950X.2014.12142.x. 24521049

[26] Hjelm-Björkman AK, Kuusela E, Liman A, Markkola A, Saarto E, Huttunen P, et al. Evaluation of methods for assessment of pain associated with chronic osteoarthritis in dogs. J Am Vet Med Assoc. 2003;222:1552–8. 10.2460/javma.2003.222.1552 12784961

[27] Hielm-Björkman AK, Rita H, Tulamo RM. Psychometric testing of the Helsinki chronic pain index by completion of a questionnaire in Finnish by dog owners of dogs with chronic signs of pain caused by osteoarthritis. Am J Vet Res. 2009;70:727–34. 10.2460/ajvr.70.6.727 19496661

[28] Hercock CA, Pinchbeck G, Giejda A, Clegg PD, Innes JF. Validation of a client-based clinical metrology instrument for the evaluation of canine elbow osteoarthritis. J Small Anim Pract. 2009. 10.1111/j.1748-5827.2009.00765.x. 19527419

[29] Clark N, Comerford E. An update on mobility assessment of dogs with musculoskeletal disease. J Small Anim Pract. 2023. 10.1111/jsap.13650. 37455329

[30] Steinert K, Kuhne F, Kramer M, Hackbarth H. People’s perception of brachycephalic breeds and breed-related welfare problems in Germany. J Vet Behav. 2019. 10.1016/j.jveb.2019.06.006.

[31] Packer RMA, Seath IJ, O’Neill DG, De Decker S, Volk HA. DachsLife 2015: an investigation of lifestyle associations with the risk of intervertebral disc disease in Dachshunds. Canine Genet Epidemiol. 2016. 10.1186/s40575-016-0039-8. PMC5097381 27826450

[32] Pulkkinen HSM, Lappalainen AK, Junnila JJT, Laitinen-Vapaavuori OM, Hyytiäinen HK. Thoracic limb angular deformity in chondrodystrophic dogs: repeatability of goniometric measurement of external rotation and carpal valgus. Vet Comp Orthop Traumatol. 2022. 10.1055/s-0042-1750030.

[33] Hall EJ, Carter AJ, Farnworth MJ. Exploring owner perceptions of the impacts of seasonal weather variations on canine activity and potential consequences for human–canine relationships. Animals. 2021. 10.3390/ani11113302. PMC8614552 34828033

[34] Finnish Kennel Club. Uusi koirakanta-arvio kertoo: Suomessa on yli 800 000 koiraa, joista yli 580 000 on Suomen Kennelliiton rekisterissä. 2023. https://www.kennelliitto.fi/tietoa-meista/uutiset/uusi-koirakanta-arvio-kertoo-suomessa-yli-800-000-koiraa-joista-yli-580-000-suomen-kennelliiton-rekisterissa. Accessed 30 Mar 2026.

[35] Kubinyi E, Turcsán B, Miklósi Á. Dog and owner demographic characteristics and dog personality trait associations. Behav Processes. 2009. 10.1016/j.beproc.2009.04.004. 19520239

[36] Anderson KL, Casey RA, Cooper B, Upjohn MM, Christley RM. National Dog Survey: Describing UK Dog and Ownership Demographics. Animals. 2023. 10.3390/ani13061072. PMC10044414 36978614

[37] Carvelli A, Scaramozzino P, Lacoponi F, Condoleo R, Della Marta U. Size, demography, ownership profiles, and identification rate of the owned dog population in central Italy. PLoS ONE. 2020. 10.1371/journal.pone.0240551. PMC7561154 33057370

[38] Stephan G, Leidhold J, Hammerschmidt K. Pet dogs home alone: a video-based study. Appl Anim Behav Sci. 2021. 10.1016/j.applanim.2021.105463.

[39] Brown DC, Michel KE, Love M, Dow C. Evaluation of the effect of signalment and body conformation on activity monitoring in companion dogs. Am J Vet Res. 2010;71:322–5. 10.2460/ajvr.71.3.322 PMC4483229 20187834

[40] Michel KE, Brown DC. Association of signalment parameters with activity of pet dogs. J Nutr Sci. 2014. 10.1017/jns.2014.49. PMC4473176 26101597

[41] Morrison R, Penpraze V, Greening R, Underwood T, Reilly JJ, Yam PS. Correlates of objectively measured physical activity in dogs. Vet J. 2013. 10.1016/j.tvjl.2013.11.023. 24378292

[42] Jimenez AG, Paul K, Zafar A, Ay A. Effect of different masses, ages, and coats on the thermoregulation of dogs before and after exercise across different seasons. Vet Res Commun. 2022. 10.1007/s11259-022-10045-2. 36449118

[43] de la Torres G, Hart BL, Farver TB, Oberbauer AM, Messam LLM, Willits N, et al. Neutering dogs: effects on joint disorders and cancers in Golden Retrievers. PLoS ONE. 2013. 10.1371/journal.pone.0055937. PMC3572183 23418479

[44] Hart BL, Hart LA, Thigpen AP, Willits NH. Long-term health effects of neutering dogs: comparison of Labrador Retrievers with Golden Retrievers. PLoS ONE. 2014. 10.1371/journal.pone.0102241. PMC4096726 25020045

[45] Hart BL, Hart LA, Thigpen AP, Willits NH. Neutering of German Shepherd Dogs: associated joint disorders, cancers and urinary incontinence. PLoS ONE. 2016. 10.1371/journal.pone.0148906. PMC5645870 29067194

[46] Graves JL, McKenzie BA, Koch Z, Naka A, Spofford N, Morrison JA. Body weight, gonadectomy, and other risk factors for diagnosis of osteoarthritis in companion dogs. Front Vet Sci. 2023. 10.3389/fvets.2023.1275964. PMC10713818 38089705

[47] Hart BL, Hart LA, Thigpen AP, Willits NH. Assisting decision-making on age of neutering for 35 breeds of dogs: associated joint disorders, cancers, and urinary incontinence. Front Vet Sci. 2020. 10.3389/fvets.2020.00588. PMC7359819 32733924

[48] Dorn M, Seath IJ. Neuter status as a risk factor for canine intervertebral disc herniation (IVDH) in dachshunds: a retrospective cohort study. Canine Genet Epidemiol. 2018. 10.1186/s40575-018-0067-7. PMC6236875 30459956

[49] Doeven L, Cardy T, Crawford AH. Investigation of neutering status and age of neutering in female Dachshunds with thoracolumbar intervertebral disc extrusion. J Small Anim Pract. 2024. 10.1111/jsap.13733. 38622029

[50] O’Neill DG, Church DB, McGreevy PD, Thomson PC, Brodbelt DC. Prevalence of disorders recorded in dogs attending primary-care veterinary practices in England. PLoS ONE. 2014. 10.1371/journal.pone.0090501. PMC3942437 24594665

[51] O’Neill DG, James H, Brodbelt DC, Church DB, Pegram C. Prevalence of commonly diagnosed disorders in UK dogs under primary veterinary care: results and applications. BMC Vet Res. 2021. 10.1186/s12917-021-02775-3. PMC7888168 33593363

[52] All-Party Parliamentary Group for Animal Welfare. Welcome to the APGAW Innate Health Assessment (IHA): the 10-point checklist for healthier dogs. 2025. https://www.innatehealthassessment.org/. Accessed 24 Nov 2025.

[53] Samoy Y, Van Ryssen B, Gielen I, Walschot N, van Bree H. Review of the literature: Elbow incongruity in the dog. Vet Comp Orthop Traumatol. 2006;19:1–8. 16594537

[54] Knapp JL, Tomlinson JL, Fox DB. Classification of angular limb deformities affecting the canine radius and ulna using the center of rotation of angulation method. Vet Surg. 2016. 10.1111/vsu.12460. 27011252

[55] Lappalainen AK, Mäki K, Laitinen-Vapaavuori O. Estimate of heritability and genetic trend of intervertebral disc calcification in Dachshunds in Finland. Acta Vet Scand. 2015. 10.1186/s13028-015-0170-7. PMC4656185 26597811

[56] Pulkkinen HSM, Reunanen VLJ, Hyytiäinen HK, Junnila JJT, Laitinen-Vapaavuori OM, Lappalainen AK. The intra- and intertester repeatability of radiographic elbow incongruity grading is high in chondrodystrophic dog breeds. Vet Radiol Ultrasound. 2020. 10.1111/vru.12853. 32141167

[57] Brown EA, Dickinson PJ, Mansour T, Sturges BK, Aguilar M, Young AE, et al. FGF4 retrogene on CFA12 is responsible for chondrodystrophy and intervertebral disc disease in dogs. Proc Natl Acad Sci. 2017. 10.3390/genes10060435. PMC5664524 29073074

[58] Bannasch D, Batcher K, Marcellin-Little DJ, Leuthard F, Bannasch M, Hug P, et al. The effects of FGF4 retrogenes on canine morphology. Genes. 2022. 10.3390/genes13020325. PMC8872533 35205370

[59] Sullivan S, Szeremeta KJ, Kutzler M. Case report: FGF4L1 retrogene insertion is lacking in the tall dachshund phenotype. Front Vet Sci. 2025. 10.3389/fvets.2024.1522745. PMC11739356 39830166

[60] Suomen Mäyräkoiraliitto. Jalostussuositukset (CDDY). https://www.mayrakoiraliitto.fi/rotutietoa/terveys/geenitestit/geenisuositukset/. Accessed 24 Jun 2026.

[61] Batcher K, Dickinson P, Giuffrida M, Sturges B, Vernau K, Knipe M, et al. Phenotypic effects of FGF4 retrogenes on intervertebral disc disease in dogs. Genes. 2019. 10.3390/genes10060435. PMC6627552 31181696

[62] Embersics C, Bannasch D, Batcher K, Boudreau EC, Church M, Miller A, et al. Association of the FGF4L2 retrogene with fibrocartilaginous embolic myelopathy in dogs. J Vet Intern Med. 2024. 10.1111/jvim.16925. PMC10800192 37916855

[63] Sullivan S, Redden D, Hardeng F, Sundqvist M, Kutzler M. The relationship between radiographic disc calcification score and FGF4L2 genotype in dachshunds. J Vet Intern Med. 2025. 10.1111/jvim.17281. PMC11665960 39715441

[64] Proschowsky HF, Arendt ML, Bonnett BN, Bruun CS, Czycholl I, Fredholm M, et al. A new future for dog breeding. Anim Welf. 2025. 10.1017/awf.2024.66. PMC11810519 39935775

[65] Priester WA. Canine intervertebral disc disease: occurrence by age, breed, and sex among 8,117 cases. Theriogenology. 1976;6:293–303.

[66] Lappalainen AK, Vaittinen E, Junnila J, Laitinen-Vapaavuori O. Intervertebral disc disease in Dachshunds radiographically screened for intervertebral disc calcifications. Acta Vet Scand. 2014. 10.1186/s13028-014-0089-4. PMC4285634 25523328

