# Supplementary material for: Exercise habits and musculoskeletal health of the Finnish Dachshund population: an online survey study
Source: Acta Vet Scand. 2026 Aug 13;68:49. doi: 10.1186/s13028-026-00877-2 (PMC13744107; doi:10.1186/s13028-026-00877-2)
Supplement: Supplementary file 1 [file 13028_2026_877_MOESM1_ESM.pdf]

Dachshund Exercise Survey 2022

The University of Helsinki, Faculty of Veterinary Medicine, Unit of Diagnostic Imaging, and the research group FaunaFysio are, with the enclosed survey, exploring the exercise habits of Finnish Dachshunds. In addition, limb positions typical for Dachshunds are examined. This survey is part of the PhD research by Kami Turunen, DVM, on Dachshund pes varus deformity.

We are seeking responses from owners of purebred Dachshunds that are at least one year old and still alive. A separate survey should be filled out for each dog that meets the participation criteria. If the dog's owner and caretaker are different people or if the dog has multiple owners, please complete the survey together so that only one survey is submitted per dog. The dog's basic information can be found in the KoiraNet Breeding Database if needed.

The survey can be completed on a mobile phone, tablet, or computer. Responding to the survey will take approximately 10-20 minutes. You can also save the survey as incomplete and finish it later, submitting it when convenient. If you're unsure about an answer, it's best to select the option that seems most accurate.

The information and answers provided by participants will be handled confidentially by the research group, and no data will be shared outside the PhD research group. The research data will be analysed and published in a way that ensures that individual respondents or dogs cannot be identified or distinguished from the results. For more information on how personal data are handled, please refer to the study's privacy statement.

The results of the study will be published in an international peer-reviewed veterinary journal and will be communicated through the Finnish Dachshund Club, the Finnish Kennel Club, and the Veterinary Teaching Hospital's communication channels and social media.

For further details about the study, please contact PhD researcher Kami Turunen, DVM (kami.turunen@helsinki.fi).

Owner's Information

I have read the survey's privacy notice, and by answering the survey, I give my consent for the use of my responses in research: Yes

My age: I am at least 15 years old

Owner's name:

Owner's email address:

Owner’s phone number:

Based on the survey, owners who are willing will be invited to participate in voluntary follow-up studies with their dogs. These studies include physiotherapeutic, orthopaedic, and diagnostic imaging examinations (CT scan and radiography of the pelvis and hind limbs under sedation). All follow-up studies will be conducted at the Veterinary Teaching Hospital (Koetilantie 2, 00790 Helsinki). Participation in the studies is free of charge for the owner.

I can be contacted regarding possible follow-up examinations:

Yes

No

Dog’s Basic Information

The dog is a purebred Dachshund:

Yes

Dog’s breed:

Kaninchen wire-haired Dachshund

Kaninchen smooth-haired Dachshund

Kaninchen long-haired Dachshund

Miniature wire-haired Dachshund

Miniature smooth-haired Dachshund

Miniature long-haired Dachshund

Standard wire-haired Dachshund

Standard smooth-haired Dachshund

Standard long-haired Dachshund

The dog is registered:

Yes

No

Dog’s registration number:

Filling in this section is optional.

You can find the dog’s registration number in the KoiraNet Breeding Database if needed.

My response may be linked to the dog's information in the KoiraNet breeding database:

Yes

No

Dog’s name:

Dog’s weight:

Weight in kilograms (kg). If you don’t know the weight, estimate it as accurately as possible.

Dog’s date of birth:

DD.MM.YYYY

Dog’s sex:

Male

Female

Neutered male

Spayed female

Is the dog pregnant or does it have puppies under adoption age?

The dog has been mated, but pregnancy is uncertain.

The dog is currently pregnant.

The dog has puppies under adoption age.

No.

How many dogs are in the household?

One

Two

Three

Four or more

Dog’s General Health

Is your dog affected by any physical issue that impairs its daily quality of life?

Yes

No

What physical issue or issues does your dog have that impair its quality of life?

List here all physical issues that impair your dog's quality of life.

Does your dog have any chronic illness or recurring signs of illness?

Yes

No

What chronic illness or recurring signs of illness does your dog have?

List here all chronic illnesses or recurring signs of illness your dog has.

Does your dog currently have any acute or new illness, injury, or condition?

Yes

No

What acute or new illness, injury, or condition does your dog have?

List here all acute or new illnesses, injuries, or conditions your dog has.

Does your dog receive any medications or treatments?

Yes

No

What medications or treatments does your dog currently receive, and what are they for?

List here the name of the medication/treatment and/or the reason for the medication/treatment.

Questions Related to Dog's Movement and Functionality

In the following sections, describe your dog's typical exercise habits. If your dog currently has an acute or new temporary illness, injury, or condition, answer the survey based on how your dog behaved before it became ill.

How much exercise does your dog get daily?

Estimate here how much your dog moves in total each day. Exercise includes, for example, walking, outdoor activity, play, and hobbies.

Less than 30 minutes per day

At least 30 minutes per day

At least 1 hour per day

At least 2 hours per day

More than 3 hours per day

How much intense exercise does your dog get daily?

Estimate here all your dog's active exercise such as energetic play, running, active hobbies, and brisk walks.

Less than 30 minutes per day

At least 30 minutes per day

At least 1 hour per day

At least 2 hours per day

More than 3 hours per day

Which of the following forms of physical play does your dog regularly engage in?

Select here all activities that your dog regularly engages in.

Running off-leash

Digging

Fetching a ball or other object

Tugging a rope or other object

Running with a toy

Wrestling with other dogs

Water play

Other

My dog does not play

If you answered "Other," please specify.

Which of the following activities do you regularly engage in with your dog?

Select here all activities that you regularly engage in with your dog.

Walking on a leash or off-leash

Running and/or Canicross

Earthdog activities

Drive hunting

Tracking

Agility

Rally obedience

Obedience

Canine freestyle

Other

If you selected "Other," please specify.

How often does your dog go out daily?

Going out refers to all time spent outdoors, including both relaxing and active exercise and walking.

My dog does not go out

Once a day

Twice a day

Three times or more

How much time does your dog spend out daily?

Going out refers to all time spent outdoors, including both relaxing and active exercise, and walking.

Less than 30 minutes per day

At least 30 minutes per day

At least 1 hour per day

At least 2 hours per day

More than 3 hours per day

Your dog's most typical daily outdoor habits:

Select here all your dog's daily outdoor habits.

On a leash

Off-leash

In a fenced yard or enclosure

On a leash line

At a dog park

What type of surface does your dog primarily walk on outdoors?

Select here all surfaces your dog regularly walks on.

Forest terrain or similar

Grass, field, or similar

Dirt road

Asphalt or similar

Other

If you selected "Other," please specify.

How many walks does your dog take daily?

A walk is defined as at least 15 minutes of brisk walking on a leash or off-leash.

My dog does not walk

One walk

Two walks

Three or more walks

How often does your dog get to run off-leash on average?

Consider off-leash running in, for example, the yard, enclosure, or forest.

Not at all

Occasionally

Weekly

Daily

Does your dog regularly use stairs?

Yes

No

In which direction does your dog move on the stairs?

Up and down

Upwards

Downwards

How many stairs does your dog typically climb each day?

Less than 30

More than 30

Why doesn't your dog use stairs?

The dog is not allowed to use stairs

There are no stairs in the dog's environment

The dog is unwilling to use stairs

Does your dog show reluctance to locomote?

Not at all

Occasionally

Weekly

Daily

What do you think is the reason for your dog's reluctance to locomote?

Does your dog have a reluctance to jump?

Consider all types of jumping here such as jumping onto the bed or sofa, into the car, or over obstacles.

Not at all

Occasionally

Weekly

Daily

Does your dog have difficulty getting up from a lying position?

Not at all

Occasionally

Weekly

Daily

Does your dog limp?

Not at all

Occasionally

Weekly

Daily

Purpose of the Dog

What was the primary intended purpose for which your dog was acquired?

Pet dog

Hunting dog

Show dog

Breeding dog

Other

If you answered "Other," what was the primary intended purpose?

Did your dog have any other intended purposes when it was acquired?

No other purposes

Pet dog

Hunting dog

Show dog

Breeding dog

Other

If you answered "Other," what was the secondary intended purpose?

Has your dog been suitable for the purpose for which it was acquired?

Yes

No

Why has your dog not been suitable for its purpose?

Questions Related to the Musculoskeletal System

Has a veterinarian diagnosed your dog with hip dysplasia (a growth disorder of the hip joint)?

Also consider hip dysplasia (FCI grade C, D, or E) identified by the Finnish Kennel Club screening tests. Hip dysplasia can affect both or just one of the hind limbs.

Yes

No

Not examined

In which hip joint has the growth disorder been diagnosed?

Left

Right

Both

Has a veterinarian diagnosed your dog with patellar luxation (dislocation of the kneecap)?

Patellar luxation can affect both or just one of the hind limbs.

Yes

No

Not examined

Has your dog been diagnosed with lateral (outward) or medial (inward) patellar luxation?

<u>Left hind limb:</u>

Lateral (outward)

Medial (inward)

No patellar luxation in this limb

I don’t know

<u>Right hind limb:</u>

Lateral (outward)

Medial (inward)

No patellar luxation in this limb

I don’t know

Has a veterinarian diagnosed your dog with a disc herniation (also known as “Dachshund paralysis”)?

Yes

No

Have you noticed any signs in your dog that could indicate a disc herniation (also known as “Dachshund paralysis”)?

Yes

No

At what age did signs indicating disc herniation first appear?

Please specify your dog's age in months and/or years.

What kind of signs did your dog show when experiencing disc herniation?

Select all signs that your dog exhibited.

Pain and/or reluctance to locomote

Weakness or stumbling of front and/or hind legs

Paralysis of front and/or hind legs

Other

Has a veterinarian diagnosed your dog with a front limb growth disorder (e.g., outward turning of the paws or elbow incongruence)?

Growth disorders can affect both or just one front limb.

Yes

No

Not examined

In which front limb has the growth disorder been diagnosed?

Left

Right

Both

What type of front limb growth disorder has your dog been diagnosed with?

Has your dog been suspected or diagnosed with pes varus (inward tilting of the hind paw and/or bow-leggedness)?

Pes varus can affect both or just one hind limb.

Yes

No

In which hind limb has pes varus been diagnosed or suspected?

Left

Right

Both

Has your dog received any treatments for front or hind limb growth problems?

Yes

No

What treatments has your dog received for front or hind limb growth issues?

Rest and/or exercise restriction

Pain medication

Physiotherapy

Surgical treatment

Other

If you selected "Other," please specify.

Has your dog undergone surgery on its front or hind limbs due to a growth disorder or another reason?

Yes

No

Which limb(s) has your dog had surgery on?

Select all limbs that have been operated.

Left front limb
Right front limb
Left hind limb
Right hind limb

Why was surgery performed on your dog’s limb(s)?

When was the surgery performed?
If you don’t remember the exact date, please provide an estimate.
D-M-Y

Questions Related to Conformation of the Dog's Limbs

Front Limb Conformation

In this section, we ask you to assess your dog’s front limb conformation using the provided image scale. Make your assessment based on what you see either when the dog is standing still or moving. Your assessment can be based on both. You may also palpate the dog’s limb conformation if necessary.
It is easiest to evaluate the standing position when the dog is standing in a balanced, symmetrical position: front limbs parallel, hind limbs parallel, head facing straight ahead, and back straight on a stable, non-slippery surface at an appropriate height such as on a table with a rubber mat.
For movement assessment, the dog should walk toward you so that you can see the front limbs directly from the front.
The left and right limbs of the dog are defined by viewing the dog from behind.

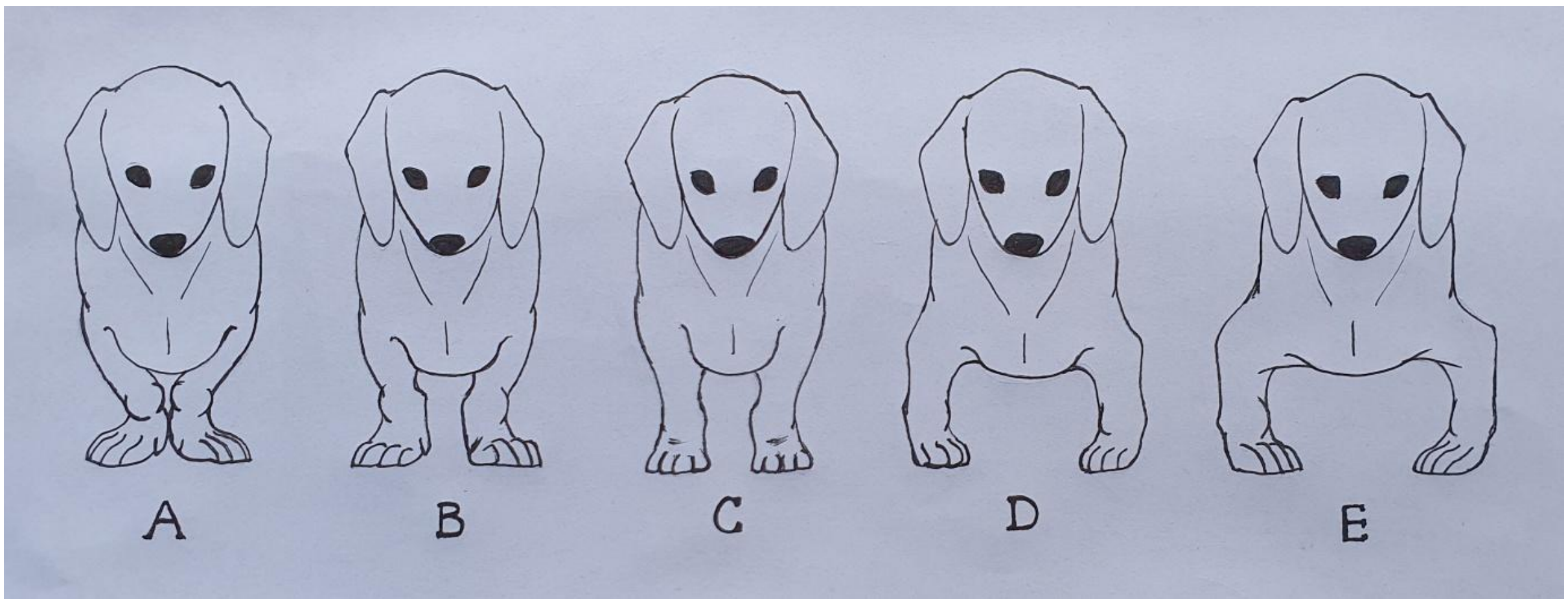

Left front limb:

A

B

C

D

E

Right front limb

A

B

C

D

E

Hind Limb Conformation

In this section, we ask you to assess your dog's hind limb conformation using the provided image scale. Make your assessment based on what you see either when the dog is standing still or moving. Your assessment can be based on both. You may also palpate the dog's limb conformation if necessary.

It is easiest to evaluate the standing position when the dog is standing in a balanced, symmetrical position: front limbs parallel, hind limbs parallel, head facing straight ahead, and back straight on a stable, non-slippery surface at an appropriate height such as on a table with a rubber mat.

For movement assessment, the dog should walk in front of you so that you can see the hind limbs directly from behind.

The left and right limbs of the dog are defined by viewing the dog from behind.

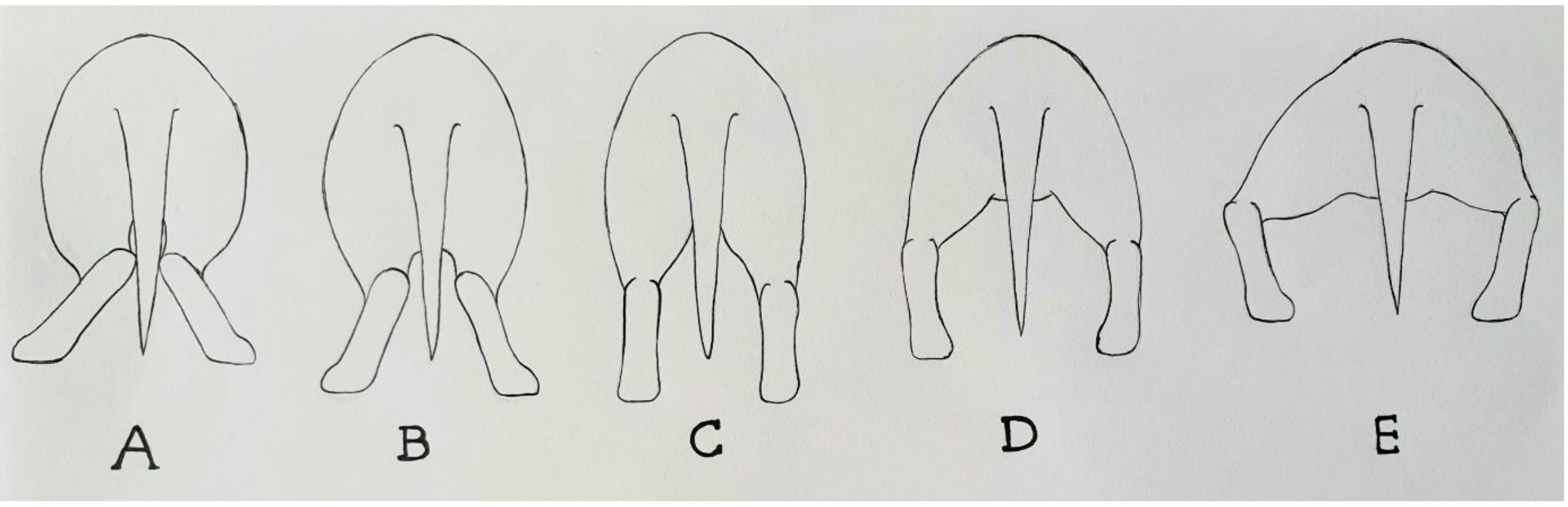

Left hind limb:

A

B

C

D

E

Right hind limb:

A

B

C

D

E

Final Remarks

Do you have anything else you would like to add to your responses?

Completing this section is optional.

Thank you for participating in the Dachshund Exercise Survey!

Submit

Save & Return Later
