# Supplementary material for: Exercise habits and musculoskeletal health of the Finnish Dachshund population: an online survey study
Source: Acta Vet Scand. 2026 Aug 13;68:49. doi: 10.1186/s13028-026-00877-2 (PMC13744107; doi:10.1186/s13028-026-00877-2)
Supplement: Supplementary file 2 [file 13028_2026_877_MOESM2_ESM.pdf]

Additional file 2. Population demographics.

Variable	Category	n	%
Age	1.0-1.3 years (P_{10}[a]=1.3)	70/640	10.9
	1.4-2.1 years (P_{25}=2.1)	101/640	15.8
	2.2-3.8 years (P_{50}=3.8)	149/640	23.3
	3.9-6.7 years (P_{75}=6.7)	161/640	25.2
	6.8-9.6 years (P_{90}=9.6)	96/640	15.0
	9.7-15.8 years	63/640	9.8
Weight	2.8-4.7 kg (P_{10}=4.7)	68/638	10.7
	4.8-5.8 kg (P_{25}=5.8)	94/638	14.7
	5.9-8.0 kg (P_{50}=8.0)	191/638	29.9
	8.1-9.6 kg (P_{75}=9.6)	128/638	20.1
	9.7-11.0 kg (P_{90}=11.0)	106/638	16.6
	11.1-15.1 kg	51/638	8.0
Breed variety[b]	Smooth-haired standard	168/640	26.3
	Wire-haired standard	141/640	22.0
	Long-haired miniature	104/640	16.3
	Smooth-haired miniature	87/640	13.6

	Wire-haired miniature	77/640	12.0
	Long-haired standard	63/640	9.8
Neuter status	Female, intact	286/640	44.7
	Male, intact	221/640	34.5
	Female, neutered	88/640	13.8
	Male, neutered	45/640	7.0
Primary intended purpose	Pet	398/640	62.2
	Hunting	108/640	16.9
	Breeding	74/640	11.6
	Show	47/640	7.3
	Other	13/640	2.0

[a] P_{10}-P_{90} denote the 10th, 25th, 50th, 75th and 90th percentiles, respectively.

[b] Both miniature and kaninchen size variations are included in miniature size.
