# Supplementary material for: Exercise habits and musculoskeletal health of the Finnish Dachshund population: an online survey study
Source: Acta Vet Scand. 2026 Aug 13;68:49. doi: 10.1186/s13028-026-00877-2 (PMC13744107; doi:10.1186/s13028-026-00877-2)
Supplement: Supplementary file 3 [file 13028_2026_877_MOESM3_ESM.pdf]

Additional file 3. Associations between number of dogs in the household and exercise habits alongside medication usage.

		95% CI		
**Effects on Exercise Score (ES)**	ES estimate[a]	Lower	Upper	P-value
Number of dogs[b]:				
1 vs. 2	-0.4154	-0.7852	-0.0455	0.028*
1 vs. 3	-0.5650	-1.0267	-0.1033	0.017*
1 vs. 4 or more	-1.0493	-1.4622	-0.6363	<.001*
2 vs. 3	-0.1497	-0.6059	0.3065	0.520
2 vs. 4 or more	-0.6339	-1.0208	-0.2470	0.001*
3 vs. 4 or more	-0.4842	-0.9362	-0.0322	0.036*

		95% CI			
**Less time spent outdoors**	Odds ratio	Lower	Upper	T-value	P-value
Number of dogs:					
1 vs. 2	1.06	0.72	1.56	0.29	0.774
1 vs. 3	1.44	0.89	2.32	1.50	0.133
1 vs. 4 or more	2.51	1.62	3.88	4.13	<.001*
2 vs. 3	1.36	0.85	2.19	1.28	0.202
2 vs. 4 or more	2.37	1.56	3.61	4.04	<.001*
3 vs. 4 or more	1.74	1.07	2.82	2.26	0.024*
**Fewer walks**[c]					
Number of dogs:					
1 vs. 2	0.67	0.45	0.99	-2.00	0.046*
1 vs. 3	0.55	0.34	0.91	-2.35	0.019*
1 vs. 4 or more	0.24	0.15	0.37	-6.31	<.001*
2 vs. 3	0.83	0.51	1.33	-0.78	0.435
2 vs. 4 or more	0.36	0.24	0.54	-4.89	<.001*

3 vs. 4 or more	0.43	0.27	0.70	-3.46	<.001*
**Less running off-leash**					
Number of dogs:					
1 vs. 2	2.35	1.56	3.54	4.10	<.001*
1 vs. 3	4.05	2.38	6.89	5.16	<.001*
1 vs. 4 or more	7.88	4.71	13.20	7.86	<.001*
2 vs. 3	1.72	1.01	2.92	2.02	0.044*
2 vs. 4 or more	3.35	2.05	5.49	4.81	<.001*
3 vs. 4 or more	1.95	1.10	3.45	2.28	0.023*
**Risk of receiving medications and treatments**					
Number of dogs:					
1 vs. 2	2.68	1.16	6.16	2.32	0.021*
1 vs. 3	0.91	0.40	2.08	-0.23	0.820
1 vs. 4 or more	2.88	1.20	6.89	2.38	0.018*
2 vs. 3	0.34	0.13	0.88	-2.21	0.027*
2 vs 4 or more	1.07	0.40	2.89	0.14	0.887
3 vs. 4 or more	3.17	1.23	8.18	2.38	0.017*

[a] Higher ES indicating more physical activity. [b] Number of dogs living in the same household.
[c] At least 15 minutes of brisk walking on a leash or off-leash. * Statistically significant result.
