# Supplementary material for: Exercise habits and musculoskeletal health of the Finnish Dachshund population: an online survey study
Source: Acta Vet Scand. 2026 Aug 13;68:49. doi: 10.1186/s13028-026-00877-2 (PMC13744107; doi:10.1186/s13028-026-00877-2)
Supplement: Supplementary file 4 [file 13028_2026_877_MOESM4_ESM.pdf]

Additional file 4. Exercise habits of the Finnish dachshund population.

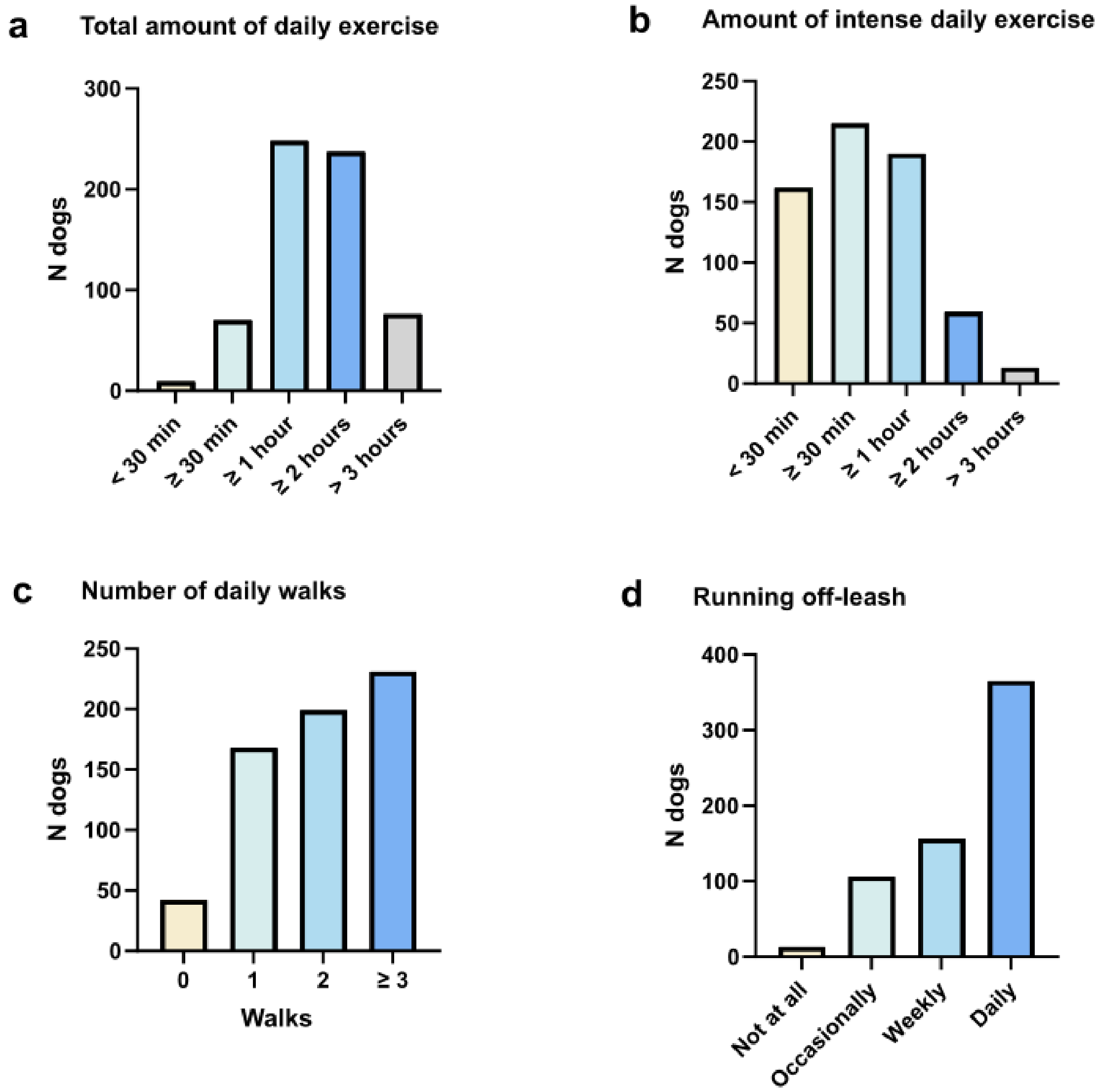

Daily amount of total (a) and intense (b) exercise. c: number of daily walks i.e. at least 15 minutes of brisk walking on or off-leash. d: graph indicates how often dogs are allowed to run off-leash.
