# Supplementary material for: Exercise habits and musculoskeletal health of the Finnish Dachshund population: an online survey study
Source: Acta Vet Scand. 2026 Aug 13;68:49. doi: 10.1186/s13028-026-00877-2 (PMC13744107; doi:10.1186/s13028-026-00877-2)
Supplement: Supplementary file 5 [file 13028_2026_877_MOESM5_ESM.pdf]

Additional file 5. Factors associated with dog's primary intended purpose.

	95% CI			
	Odds ratio	Lower	Upper	P-value
**Breeding dog compared to Pet dog**				
Any malposition[a]: Yes vs. No	0.50	0.29	0.88	0.016*
Size variant: Standard vs. Miniature	1.38	0.49	3.88	0.543
Sex: Male vs. Female	0.65	0.32	1.33	0.238
Neuter status: Intact vs. Neutered	2.44	1.10	5.40	0.027*
Number of dogs[b]:				
Single vs. Multiple	0.45	0.23	0.87	0.018*
2 vs. 1	7.81	1.71	35.71	0.008*
3 vs. 1	11.20	2.34	53.54	0.003*
4 or more vs. 1	52.43	12.17	225.9	<.001*
2 vs. 3	0.70	0.28	1.74	0.439
2 vs. 4 or more	0.15	0.07	0.31	<.001*
3 vs. 4 or more	0.21	0.10	0.47	<.001*
**Hunting dog compared to Pet dog**				
Any malposition: Yes vs. No	1.01	0.62	1.65	0.975

Size variant: Standard vs. Miniature	19.23	6.55	56.50	<.001*
Sex: Male vs. Female	2.38	1.32	4.29	0.004*
Neuter status: Intact vs. Neutered	2.41	1.20	4.84	0.014*
Number of dogs:				
Single vs. Multiple	0.35	0.20	0.60	<.001*
2 vs. 1	2.09	1.03	4.21	0.041*
3 vs. 1	4.68	2.11	10.35	<.001*
4 or more vs. 1	12.22	5.89	25.36	<.001*
2 vs. 3	0.45	0.21	0.94	0.034*
2 vs. 4 or more	0.17	0.09	0.33	<.001*
3 vs. 4 or more	0.38	0.19	0.77	0.008*
**Show dog compared to Pet dog**				
Any malposition: Yes vs. No	0.40	0.21	0.77	0.006*
Size variant: Standard vs. Miniature	3.28	1.00	10.81	0.051
Sex: Male vs. Female	1.91	0.92	3.99	0.083
Neuter status: Intact vs. Neutered	1.38	0.60	3.18	0.446
Number of dogs:				
Single vs. Multiple	1.08	0.51	2.28	0.8364

2 vs. 1	4.34	1.52	12.43	0.006*
3 vs. 1	1.65	0.37	7.35	0.510
4 or more vs. 1	12.34	4.33	35.15	<.001*
2 vs. 3	2.63	0.72	9.67	0.145
2 vs. 4 or more	0.35	0.17	0.75	0.006*
3 vs. 4 or more	0.13	0.04	0.47	0.002*

Odds ratios for differences between groups when other purposes are compared to Pet dogs. [a] Carpal and/or tarsal varus and/or valgus malposition in one or more limbs. [b] Number of dogs living in the same household. [*] Statistically significant result.
